# Deletion of the small GTPase *rac1* in *Trichoderma reesei* provokes hyperbranching and impacts growth and cellulase production

**DOI:** 10.1186/s40694-019-0078-5

**Published:** 2019-10-18

**Authors:** Elisabeth Fitz, Christian Gamauf, Bernhard Seiboth, Franziska Wanka

**Affiliations:** 1 Research Division Biochemical Technology, Institute of Chemical, Environmental & Bioscience Engineering, TU Wien, Vienna, Austria; 2 Austrian Centre of Industrial Biotechnology (ACIB) GmbH c/o Research Division Biochemical Technology, Institute of Chemical, Environmental & Bioscience Engineering, TU Wien, Vienna, Austria; 3 Group Biotechnology, Clariant Produkte (Deutschland) GmbH, Planegg, Germany

**Keywords:** *Trichoderma reesei*, Strain engineering, Hyperbrancher, Rac, Rho GTPase, Apolar growth, Cellulase, CEL12A, Viscosity

## Abstract

**Background:**

*Trichoderma reesei* is widely known for its enormous protein secretion capacity and as an industrially relevant producer of cellulases and hemicellulases. Over the last decades, rational strain engineering was applied to further enhance homologous and heterologous enzyme yields. The introduction of hyperbranching is believed to increase protein secretion, since most exocytosis is located at the hyphal apical tip. There are several genetic modifications which can cause hyperbranching, for example the deletion of the small Rho GTPase *rac*. Rac plays a crucial role in actin dynamics and is involved in polarisation of the cell during germination and apical extension of the hyphae.

**Results:**

We deleted *rac1* in a *T. reesei* strain with an ectopically overexpressed endoglucanase, CEL12A, under P*cdna1* control. This deletion provoked a hyperbranching phenotype and strong apolar growth during germination and in mature hyphae. The strains displayed dichotomous branching and shorter total mycelium length with a larger hyphal diameter. Δ*rac1* strains exhibited a decreased radial growth on solid media. Biomass formation in liquid cultures was carbon source dependent; similar to the reference strain during growth on lactose, increased on d-glucose and slightly enhanced on cellulose. While extracellular cellulase activities remained at parental strain levels on d-glucose and cellulose, the specific activity on lactose cultures was increased up to three times at 72 h accompanied by an upregulation of transcription of the main cellulases. Although the morphology of the Δ*rac1* strains was considerably altered, the viscosity of the culture broth in fed-batch cultivations were not significantly different in comparison to the parental strain.

**Conclusions:**

Deletion of the small Rho GTPase *rac1* changes the morphology of the hyphae and provokes hyperbranching without affecting viscosity, independent of the carbon source. In contrast, biomass formation and cellulase production are altered in a carbon source dependent manner in the Δ*rac1* strains.

## Background

*Trichoderma reesei* is an industrial producer of cellulases and hemicellulases and a model organism for plant biomass degradation. Its potential for recombinant protein production lies within its high protein secretion capacity for cellulases which reaches up to 100 g per litre and its ability to grow on cheap lignocellulosic materials [1, 2]. While native cellulase production is induction dependent and can be activated by carbon sources like cellulose, cellulosic materials, lactose; and abolished on d-glucose [3]. Rational strain engineering to optimise and enhance protein production is of substantial industrial interest, since the capacity for protein secretion is high but yields for heterologously expressed proteins are often only low or moderate [4]. Tools to influence macro-morphology and morphological engineering can be valuable for optimising the production of metabolites and proteins. Modulation of macro-morphology is widely established for other industrially used filamentous fungi such as *Aspergillus* species [5–7], whereas the macro-morphology of *T. reesei* has been addressed in only a few studies so far [8, 9]. Usually, two main forms of macro-morphology are described in submerged cultures, hyphal pellets and freely dispersed mycelium. Two modes of action for how biomass agglomerations are formed are known, coagulative and non-coagulative agglomeration types [10, 11]. In the coagulative agglomeration type, the conidiospores agglomerate, in the non-coagulative type, the hyphae agglomerate after the spores have already germinated. However, often filamentous fungi can show both forms depending on the cultivation conditions [11, 12]. Pellet formation is associated with higher branching frequencies compared to dispersed mycelium [13, 14]. The optimal production morphology depends on the desired product and both macro-morphologies have their advantages and disadvantages. Pellets are not supplied evenly due to the poorer accessibility of nutrients, whereas long unsheltered hyphae of dispersed mycelium are less resistant to shear stress. The macro-morphology influences the viscosity of the broth [15], which can, in turn, affect supply of the fungus with nutrients by making even distribution through stirring harder. A compact but still dispersed growth could decrease viscosity [16], though there are no clear indicators for predicting viscosity changes.

It is widely accepted that most of the proteins are secreted from the hyphal tip during apical growth of the hyphae [13, 17, 18]. Additionally, some studies also found secretion at the septa [19–21]. The question arises, if a hyperbrancher could increase protein production by increasing the number of tips. Several studies were conducted to find a correlation between the number of tips and the protein secretion—with contradictory results. Some found a positive correlation [7, 22], some determined no correlation [13, 16, 23, 24]. The secretion pathway was subject of many studies, but our understanding is still incomplete [25]. Roughly, extracellular proteins are translocated to the endoplasmic reticulum (ER), where they are folded and glycosylated before they are packed in vesicles and transported to the Golgi apparatus. After further modifications, the proteins are transported in vesicles towards the plasma membrane and released to the exterior of the cell [26–28]. Protein secretion is influenced by many factors e.g. the capacity of the ER, the cell internal redox state, the carbon source, the growth phase, the target protein and maybe also by the hyphal architecture [29]. On a genetic level, the regulation involves a range of proteins, among them small GTPases of the Ras superfamily including Rho, Cdc42 and Rac. Those signal transduction proteins are not only involved in vesicle trafficking, they also play a crucial role in the polarisation of the cell [30, 31], especially regarding actin and microtubule dynamics [32].

Hyperbranching can be a consequence of a number of different mutations, e.g. the deletion of *vel1* in *T. reesei*, where the enhanced number of tips was accompanied by a reduced growth rate, loss of conidiation and impairment of cellulase and hemicellulase expression on inducing carbon sources [33]. Most frequently, it is a consequence of dichotomous branching (besides lateral branching) introduced by deletion or repression of genes coding for actin, formin, polarisome components or certain Rho GTPases [23]. The hyperbranching is provoked by direct or indirect perturbation of actin assembly and the interlinked polarised growth of the cell. A transversion of the *act1* reading frame in *Neurospora crassa* impaired actin assembly at the apical tip and produced a hyperbrancher phenotype, presumably due to meddling with Ca^2+^-signalling and vesicle trafficking, respectively [34]. The deletion of the formin SepA in *Aspergillus nidulans* caused a temperature sensitive hyperbrancher, depolarised growth and perturbed septa formation [35, 36].

The deletion of the small GTPase *racA* was found to produce a hyperbranching phenotype in *A. niger* without reduced biomass formation [23, 37]. Kwon et al. [23] showed that deletion of *racA* and the associated hyperbranching had no effect on the native protein production of *A. niger*, on the other hand, Fiedler et al. [38] established a production platform for *A. niger*, in which the deletion of *racA* enhanced protein secretion of the overexpressed glucoamylase. In both studies the hyperbranching was accompanied by apolar growth of the hyphae and a reduced ability to form pellets. In *A. niger*, *racA* is mainly present at the apical tip of growing hyphae, especially during germination [37]. Interestingly, the dominant activation of RacA, led to an altered morphology due to actin localisation defects [23]. Similar hyperbranching phenotypes for *rac* deletion strains were found in *A. nidulans* [32], *N. crassa* [39] and *Penicillium marneffei* [40, 41].

The aim of this study was to morphologically engineer *T. reesei* by deletion of its *racA* homologue and to characterize the deletion strains with regard to their altered morphology, the effect on native and recombinant cellulase production and viscosity properties in bioreactor cultivations. Therefore, the endoglucanase CEL12A was put under control of the constitutive promoter of *cDNA1*, which enables monitoring of cellulase production on the repressing carbon source d-glucose [42, 43].

## Results

### Identification and deletion of the *A. niger racA* homologue in *T. reesei*

The *A. niger* Rho GTPase RacA encoded by An11g10030 [37] was used as query in a blastp search in the NCBI database to identify the *T. reesei* RAC1 encoded by the gene tre47055 (query cover 97%, E value 1e−111, identities 77%). The next most similar protein encoded in the *T. reesei* genome is a homologue of Cdc42 (query cover 95%, E value 2e−91, identities 65%). Prior to the deletion of *rac1* we introduced an overexpression cassette for CEL12A under P_*cDNA1*_ control in the *T. reesei* QM9414 Δ*tku70* strain to be able to measure cellulase production also during growth on d-glucose [43]. This construct was randomly integrated and one strain, with similar endoglucanase activity and biomass formation to a *T. reesei* QM9414 *cel12a*^+^ strain [43], was selected as our reference strain *T. reesei* K1. *Rac1* was knocked-out in K1 and 14 of 20 PCR screened transformants were found to be deleted.

### Morphology of *T. reesei* ∆*rac1* strains

**Fig. 1:**
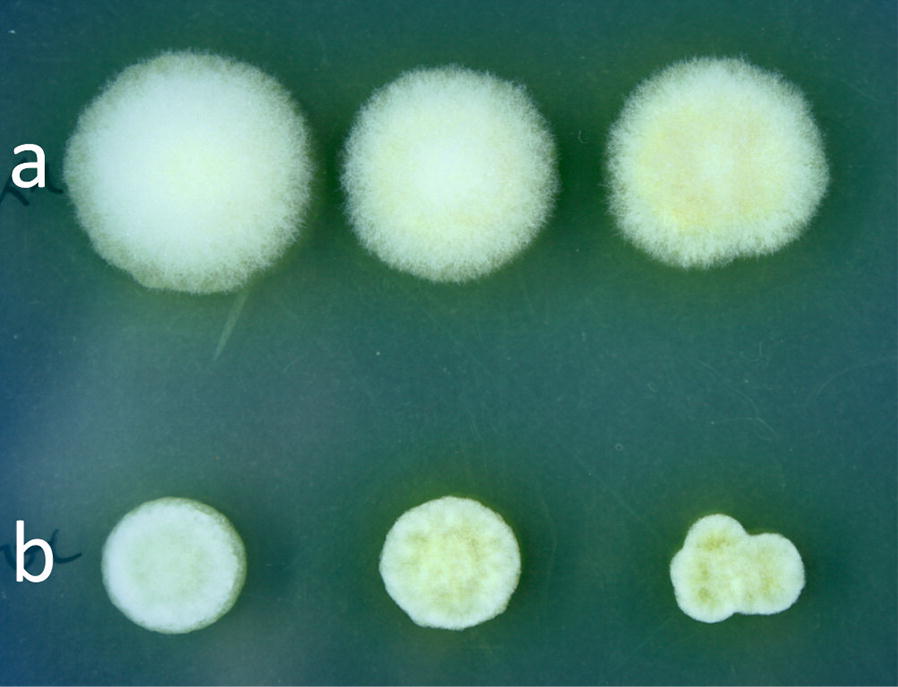
Colony morphology of *T. reesei* K1 and a representative Δ*rac1* strain on potato dextrose agar plate. A serial dilution of 10^4^ to 10^2^ spores of the reference strain K1 (**a**) and ∆*rac1* (**b**) was applied on PDA plates containing 0.1% Triton X-100 and incubated for 72 h at 28 °C

**Fig. 2:**
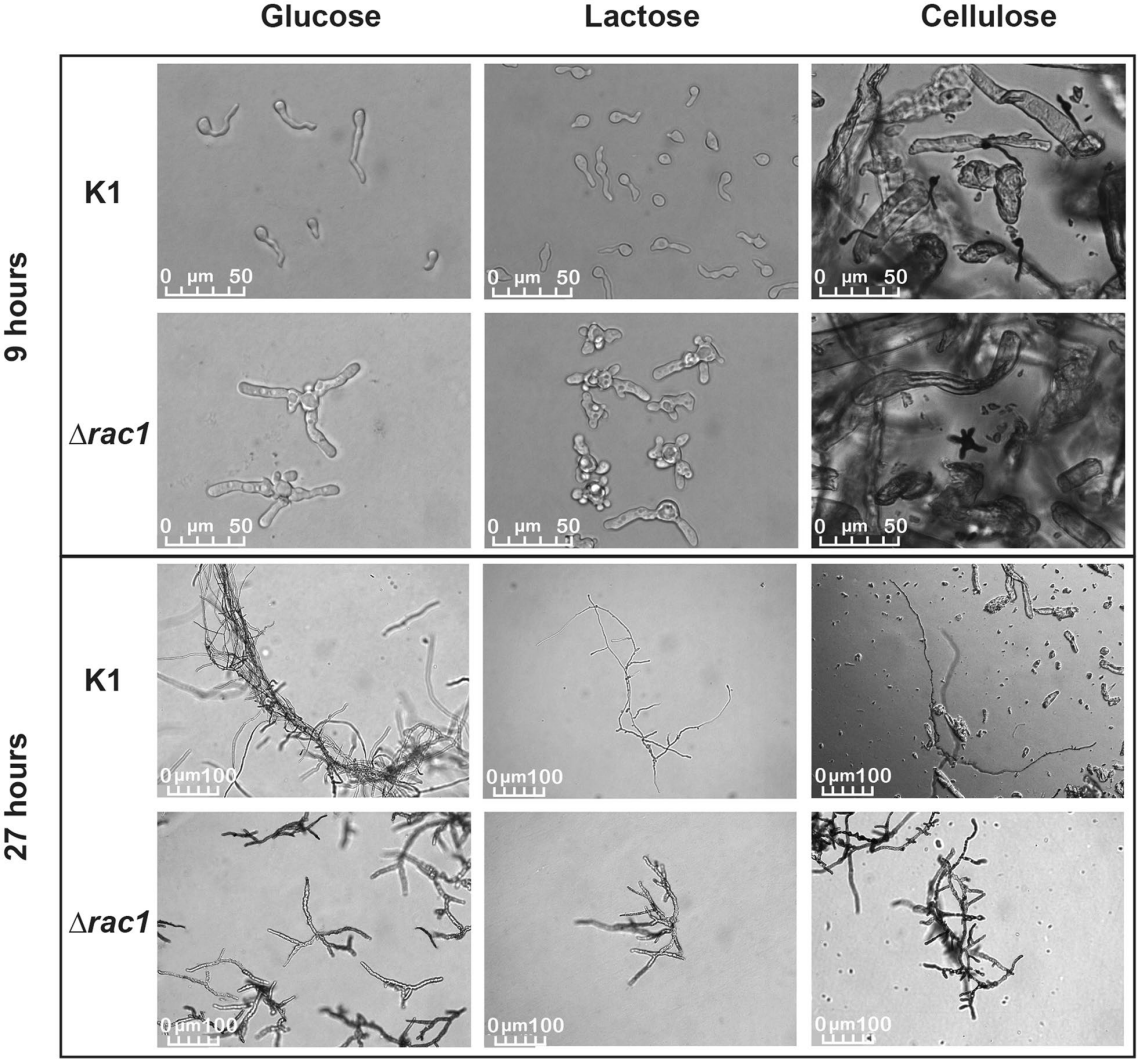
Morphological phenotypes of *T. reesei* K1 and Δ*rac1* strains in liquid cultures on different carbon sources. Δ*rac1* strains were compared to the cellulase overexpressing reference K1 at 9 and 27 h in shake flask cultivations on d-glucose, lactose and cellulose. At 9 h the spores were germinating in the shake flasks. At 27 h the mycelium was matured. The mycelium in the cellulose cultures was stained with cotton blue for enhancing the contrast

During growth on solid medium all 14 *∆rac1 T. reesei* strains exhibited an impaired, more compact radial growth with more aerial mycelium in comparison to the K1 reference strain, as depicted in Fig. 1. ∆*rac1* colonies had sharp edges, whereas colonies of the reference strain K1 showed regular growth with fringed edges. Subsequently, the strains were grown in liquid cultures on the cellulase repressing carbon source d-glucose and the two cellulase inducing carbon sources lactose and cellulose. Samples were drawn at different time points to investigate the influence on *rac1* loss on the fungal macro-morphology (Fig. 2). The Δ*rac1* strains showed strong apolar growth on all tested carbon sources, especially visible during the germination stage. After 9 h of incubation the spores of the Δ*rac1* strains were swollen and many extension points on the spore surface were formed, seemingly not orientated to a polarisation axis. Noticeably, not all of those germ tubes formed filamentous branches. The pictures in Fig. 2 at 27 h represent mature mycelium. On all carbon sources the central hyphae of the deletion strains were shorter with a thicker diameter, more apical tips and longer branches. Furthermore, the mycelium of those strains seemed to be more dispersed than the reference strain and did not collapse into biomass clumps. The evaluation of the morphological characteristics of the mature hyphae after 27 h of growth on lactose as carbon source is summarised in Table 1. Although the morphology is affected in the ∆*rac1* strains, regular septa formation could be observed when stained with calcofluor white (data not shown).

**Table 1:** Comparative image analysis with Image J of hyphal morphologies from *T. reesei* ∆*rac1* strains compared to reference strain K1 during growth on lactose for 27 h, mean values and standard deviation are given

	K1	Δ*rac1*
Mycelium length (µm)	650.4 ± 189.5	467.3 ± 208.9
Central hyphal length (µm)	498.8 ± 128.0	214.4 ± 80.3
Average branch length (µm)	56.0 ± 28.4	70.7 ± 36.5
Number of hyphal apices	4.1 ± 1.3	5.4 ± 2.0
Diameter (µm)	4.3 ± 1.5	6.4 ± 0.4

n = 25 per strain, samples of three different biological replicates were drawn from liquid cultures after 27 h, all values shown were tested with Student’s-*t*-test p < 0.05 and were all significantly different for K1 and ∆*rac1* strains

### Growth and cellulase secretion of *T. reesei* ∆*rac1* strains

**Fig. 3:**
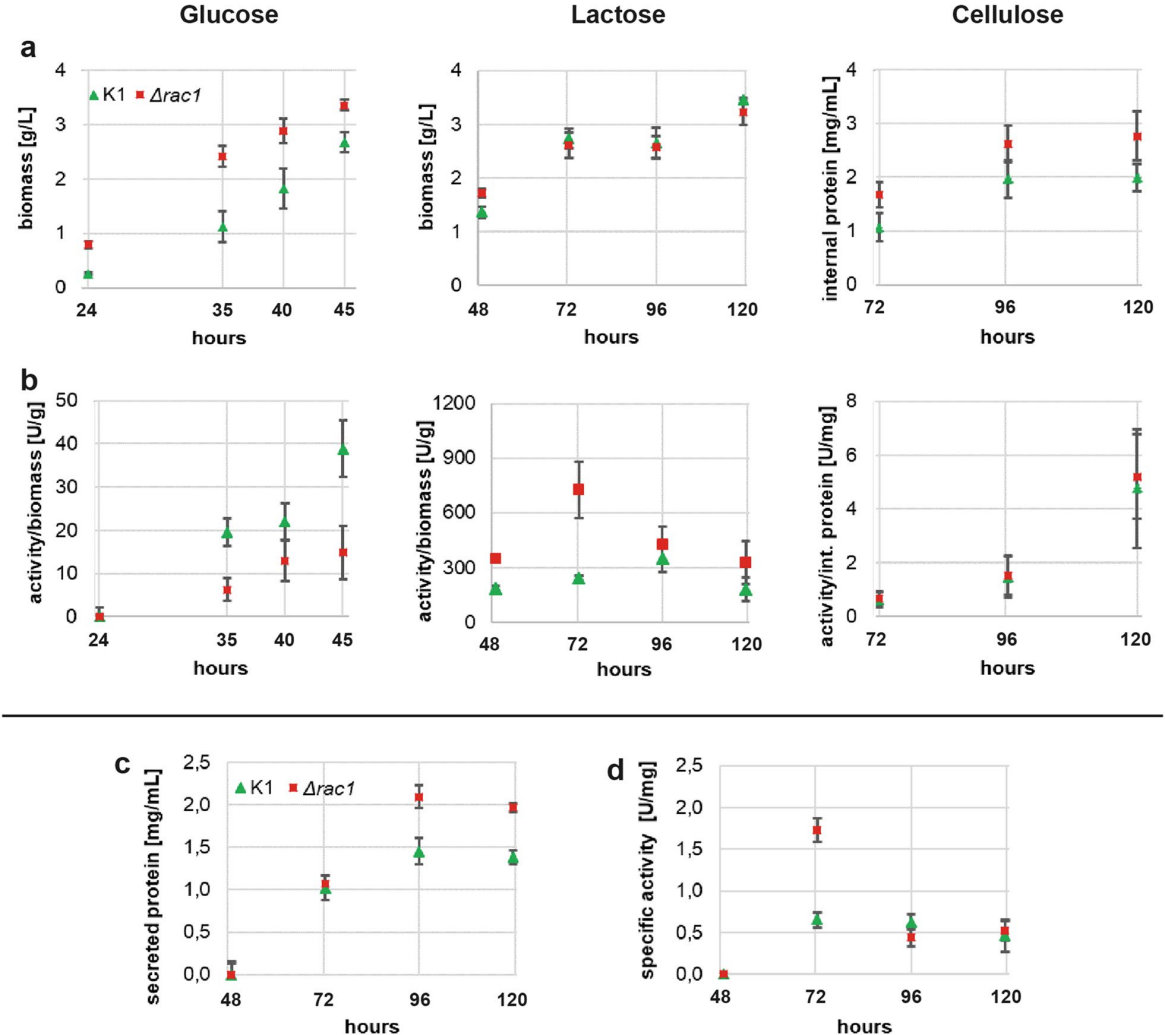
Growth, protein secretion and cellulase activity measurements of *T. reesei* K1 and Δ*rac1* liquid cultivations on different carbon sources. Five independently generated ∆*rac1* strains and three biological replicates of the K1 strain were used for all liquid culture experiments. **a** Biomass formation on d-glucose, lactose and cellulose. Since cellulose is an insoluble carbon source, biomass formation is represented by the amount of internal protein. **b** Cellulase activity per biomass. Activity in the supernatants was determined by Azo-CMC liquid assay and was related to the amount of biomass. **c** Total protein in lactose supernatants determined by a Bradford assay. **d** Specific activity of lactose supernatant, the cellulase activity was plotted against the amount of total protein in the supernatant

Since radial growth on solid media was impaired in all *T. reesei* ∆*rac1*, we tested the biomass formation in liquid cultures. Depending on the carbon source, the deletion of *rac1* resulted in different biomass formation. As depicted in Fig. 3a, the biomass formation was enhanced for Δ*rac1* strains on d-glucose compared to the reference strain K1. The biomass accumulation was twice as high at earlier time points. On cellulose the biomass, represented by the amount of internal protein, was also enhanced at all time points. Although the effect size was small, the values were confirmed to be significantly different with a t-test (p < 0.05). Interestingly, on lactose the biomass formation was the same as for the reference strain.

For further characterization, the cellulase activities of the supernatants were monitored (Fig. 3b). Lactose and cellulose are inducing carbon sources and activate native cellulase expression as opposed to it’s repression on d-glucose. In the latter case, the cellulase activity depends solely on CEL12A overexpression, which is under the control of the *cdna1* promoter and therefore independent of carbon source induction [42]. For cellulose, the volumetric cellulase activities of the supernatants of the deletion strains were in the same range as for the reference strain K1. Due to the small effect size, the ratios of both strains were equal on cellulose. For d-glucose cultures the volumetric cellulase activity resulting from the CEL12A expression were similar but, due to the increased biomass formation a reduced activity per biomass ratio was found. In contrast, on lactose higher cellulase activities were observed, peaking at 72 h at about three times the level of the reference strain K1. Notably, the total protein content in the supernatants in the lactose cultures was similar for either strains at 72 h (Fig. 3c). In conclusion, more active cellulases per total secreted protein were present in Δ*rac*1 cultures (Fig. 3d). Additionally, ∆*rac1* strains accumulated significantly more secreted proteins at the end of the shake flask cultivation.

### Loss of *rac1* leads to increased cellulase transcript levels during growth on lactose

Increased cellulase activity in lactose cultures at 72 h raised the question; whether the secretion of proteins present in the cells is more efficient, or if the expression of the cellulases is enhanced as well. Therefore, transcript levels of the major cellulase *cel7a* (*cbh1*) and *cel12a* were examined at 48 and 72 h on lactose by qPCR. In addition, we tested the expression of the two housekeeping genes *tef1* (encoding translation elongation factor) and *sar1* (encoding an ARF family GTPase) as internal reference genes. Transcript levels for both were consistent, and *sar1* was chosen for normalization.

**Fig. 4:**
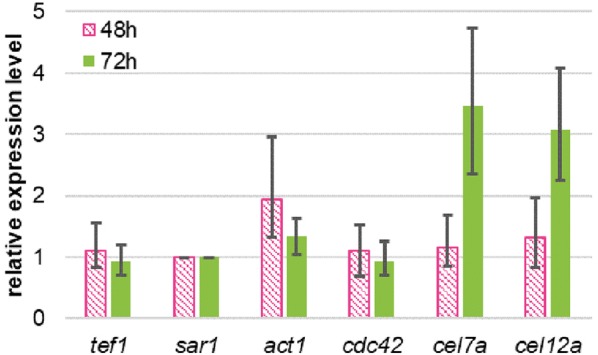
Relative mRNA transcript levels of selected genes of *T. reesei* Δ*rac1* in relation to parental K1 strains. Strains were grown for 48 and 72 h on lactose as carbon source. Three technical replicates of K1 and three biological replicates of *Δrac1* strains were grown for 48 and 72 h on lactose as carbon source. All expression data were normalized to *sar1*, then Δ*rac1* data was related to K1 values at the same time points

The transcriptomic data showed an increase of expression of both monitored cellulases (Fig. 4). The expression levels of *cel7a* were about three times higher compared to the reference strain K1. The upregulation of *cel12a* was in the same range as *cel7a* although *cel12a* transcripts can also originate from the overexpression under P*cnda1* control. Since members of the Cdc42 small GTPases share a high sequence identity and some overlapping functions with Rac proteins [31], we tested if a *rac1* deletion might influence its expression. Interestingly, the expression of the monitored *cdc42* homologue in *T. reesei* was not affected at all. Also, the expression of actin was not significantly altered, despite the drastic morphological changes and the possible actin assembly disturbance at the hyphal tips.

### Viscosity in fed-batch cultivation is not affected by *rac1* deletion

**Fig. 5:**
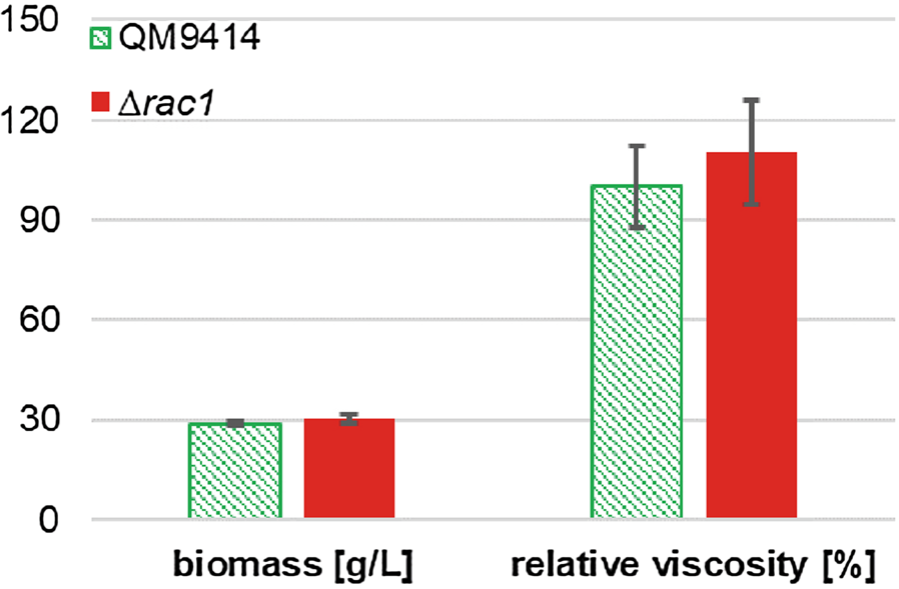
Biomass and viscosity of fed-batch cultures from *T. reesei* QM9414 and Δ*rac1*. The batches were started with d-glucose, starved for 1 h and then fed with lactose. Samples were taken after 100 h of lactose feeding. The measured angular velocities were integrated and normed to *T. reesei* QM9414

To investigate whether the altered morphology induced by hyperbranching would have an impact on the viscosity of the cultivation broth, fed-batch cultivations were performed. The strains were cultivated in a fed-batch started with d-glucose and fed with lactose. In Additional file 1: Figure S1 the process data of all twelve fed batch cultivations are illustrated. Similar to the shake flask cultivations, the biomass formation was the same for Δ*rac1* strains and the reference strain during growth on mainly lactose (Fig. 5). Although the morphology of ∆*rac1* strains was strongly affected and a slight tendency of viscosity increase can be observed in the data, there were no statistically significant differences between the deletion and reference strains, as shown in Fig. 5.

## Discussion

### Everything comes at a price: ∆*rac1* is a hyperbrancher but loses polar growth

The *T. reesei rac1* deletion strains grew in a highly apolar manner from spore to hyphae, similar to the effects observed in other filamentous fungi [23, 32, 37, 39–41]. Usually, following the activation of the dormant spore, materials for cell expansion are deposited at the cortex while a polarisation axis is established [44]. Actin cables polymerise at a defined spot and a germ tube emerges, until finally the first septum is initiated, forming the first filament. Typically, branches are formed subapically and oriented on a new polarisation axis [32, 45]. In *T. reesei* Δ*rac1* strains, the spores seemingly could not establish a proper polarisation axis and randomly formed germ tubes across the surface. Microscopic analysis showed that the visible organelles of mature hyphae seem to be “swollen”, which is a symptom of failed actin cable formation and insufficient localised transport to the apical tip [32, 46]. The actin cable formation is regulated, among others, by the small Rho GTPases, especially Rac plays a role in regulation of the actin polymerisation [31, 47] and Rac localises actin nucleation at the cell periphery like the apical tip [48]. Treatment of *A. nidulans* with anti-actin drugs provoked a similar swelling of the hyphal apex and a stop of exocytosis directed to the tip [49–51]. Since components like chitin protofilaments and glucans are transported via the cell internal microtubule and actin skeleton [52], cell wall components like hydrophobins, manno-proteins, and polysaccharides may not be effectively deposited at the tip and accumulate in the absence of Rac. This observation is in line with the suggestion by Momany [44], that in a hyperbrancher, cell wall materials are faster produced than transported to the apical tip or the cell wall. Furthermore, Rac introduces branches to the actin filaments by activation of the Arp2/3 complex [53], as does Cdc42. Although both are involved in the activation of the Arp2/3 complex over different signalling pathways, Rac by WAVE-family proteins [54] and Cdc42 by WASP-family proteins [55], the *T. reesei cdc42* could not compensate the defect caused by the *rac1* deletion.

### Changes without changes: altered morphology while viscosity unaffected

Unbranched, parallelly organised actin filaments that form filopodia-like structures can sense the extracellular matrix and also the surfaces of other cells [56]. When actin assembly and therefore the formation of filopodia-like structures is disrupted, the hyphae may lose their ability to adhere to one another and to surfaces. This could explain the observed reduced agglomeration of the hyphae, which may already start at the germinating spores. Despite the morphological changes, our data suggest that the viscosity of the cultivation broth did not change significantly. The tendency of the shorter and more compact mycelium to disperse more evenly in the medium was proposed to be a possibility to improve viscosity in the cultivation process [16]. However, there is no model to predict viscosity changes related to morphological changes so far, therefore, it is necessary to perform in vivo measurements.

### Increased cellulase production of Δ*rac1* strains only on lactose

Interestingly, the increased cellulase secretion of Δ*rac1* strains compared to the parental strain was specific for lactose and included an upregulation of the native cellulase *cel7a* and *cel12a* which is present also as a reporter in this strain under control of the *cDNA1* promoter. No improvement of cellulase secretion was found during growth on cellulose which is similar to the result found for *A. niger* on native protein production [23], although no additional overexpressed reporter was employed in the *A. niger* study. In a follow-up study, Fiedler et al. [38] found that secretion of the glucoamylase GlaA, which was put under the control of the tuneable Tet-on system in a glucoamylase deleted strain, could be enhanced in an *A. niger* Δ*racA* strain whereas the total amount of secreted enzymes stayed the same. They speculated that the secretion of non-essential cargo was increased by the hyperbrancher and that this is balanced by a feed-back mechanism termed RESS (repression under secretion stress) [57, 58]. As a consequence the overloading of the secretion pathway in the ER leads to an activation of the Unfolded Protein Response (UPR). Within a limited range, the UPR can actually improve protein production by enhancing the ER capacity for glycosylation and resulting in a faster throughput [59, 60]. In *T. reesei*, RESS leads to a selective down-regulation of genes encoding cellulases via their native promoters [58]. Therefore, it remains unclear how the observed increase in transcription levels for *cel7a*, whose product CEL7A constitutes about 60% of the *T. reesei* secretome, and *cel12a* can be explained.

### *Trichoderma reesei*, picky when it comes to protein expression

Interestingly, expression and secretion of a heterologous protein in *T. reesei* can differ from native cellulases [61, 62]. Nykänen et al. overexpressed a barley cysteine endopeptidase (EPB) and found differences in expression level, secretion capacity, and localisation in comparison to the native CBH1. Whereas EPB was only found at the apical tips, CBH1 was localised over the plasma membrane suggesting that the secretion of the foreign protein is limited to certain areas like the apical tip, whereas for native enzymes other secretory pathways are possible [19, 63]. Knowing that, it would be interesting to see whether the effect found on lactose could be repeated with a heterologous reporter. However, in this study we confirmed that an increased number of branches does not mandatorily correlate to a change in protein secretion.

## Conclusions

*Rac1* is not essential to *T. reesei* and the deletion caused apolar growth resulting in a hyperbranching phenotype. The hyperbranching drastically changed the morphology of the fungus and cellulase activity was enhanced three times during growth on lactose. Growth on d-glucose and cellulose did not trigger an increase in cellulase protein secretion, however, the deletion did not decrease production either. The introduction of stronger expression systems may support overproduction of enzymes on those carbon sources as well. The less agglomerated germinating spores and mycelium, plus the unchanged viscosity, may serve as a valuable platform for further genetic optimisations. The effect of *rac1* deletion on vesicle transport, cell wall composition, crosslinking, actin dynamics, and the polarity of the cell wall remain interesting questions for further studies.

## Materials and methods

### Strains and culture conditions

The parental strain throughout the study was *T. reesei* QM9414 Δ*tku70* [64] and it was used to construct the reference strain K1 and the Δ*rac1* deletion strains. The strains were grown on potato dextrose agar (PDA, Difco) at 28 °C. For shake flask cultivations spores were harvested with a NaCl-Tween solution (8.5 g/L NaCl, 0.9 g/L Tween-80) from PDA plates and the concentration was determined with a spectrometer at OD600. 50 mL Mandels-Andreotti medium (1.4 g/L (NH_4_)_2_SO_4_, 2 g/L KH_2_PO_4_, 0.3 g/L MgSO_4_, 0.3 g/L CaCl_2_, 0.3 g/L urea, 1 g/L peptone, 10 g/L carbon source, 20 mL/L trace elements pH 5.8 (5 mg/L FeSO_4_*7 H_2_O, 1.6 mg/L MnSO_4_*H_2_O, 1.4 mg/L ZnSO_4_*H_2_O and 2 mg/L CoCl_2_*2 H_2_O), pH adjusted to 5.5) in 250 mL flasks were inoculated with a final concentration of 10^6^ spores/mL and incubated at 28 °C in a rotary shaker at 250 rpm. Liquid cultures were grown with d-glucose, lactose or Avicel (cellulose) as carbon source. Lactose cultures were additionally supplemented with 0.5 g/L Tween-80. Liquid cultivations were performed in three technical replicates of the K1 strain and five individual Δ*rac1* strains.

*Escherichia coli* Top10 (Clontech) were used for plasmid construction and amplification. *E. coli* was grown in lysogeny broth medium (5 g/L peptone, 10 g/L yeast extract, 5 g/L NaCl) containing 100 µg/mL ampicillin.

### Vector construction

All primers and their sequences are listed in Additional file 2: Table S1. The *cel12a* overexpression vector pK1 is based on pLH_hph [65]. A 1000 bp promotor region of *cdna1* was amplified from *T. reesei* QM9414 genomic DNA by PCR using oligonucleotides Pcdna1_fw and _rv. The fragment was inserted into an *Xho*I/*Cla*I digested pLH_hph vector. *Cel12a* coding and terminator region were amplified from genomic DNA using oligonucleotides cel12a_fw and _rv. The pLH_hph_Pcdna1 vector was linearized by *Cla*I digestion and the *cel12a* PCR fragment was inserted. For change of the selection marker, the plasmid was amplified by the primers Inf_pK1_NtR_fw and _rv without the hygromycin B resistance cassette. The nourseothricin cassette was amplified using standard M13 primers from vector pBM_nat1. In this vector the dominant nourseothricin resistance marker *nat1* from *Streptomyces noursei* is under control of the *T. reesei pgi1* (encoding phosphoglucose isomerase) promoter region (Benjamin Metz, Robert H. Bischof and Bernhard Seiboth, unpublished results). The PCR fragments were fused with the NEBuilder HF DNA Assembly Kit (NEB) resulting in the final vector pK1 (pLH_Pcdna1_cel12a_nat1).

The *rac1* deletion vector was cloned in two steps: A pUC19 vector (Clontech Inc.) was opened by *Bam*HI digest and a 4.9 kb PCR amplicon of *rac1* including promoter, coding region and terminator was introduced by recombinational cloning using the NEBuilder HF DNA Assembly Kit. In a second step, the coding region of *rac1* was removed by PCR and the hygromycin B resistance cassette, amplified from pLH_hph, was inserted between the *rac1* promoter and terminator region.

PCR fragments were gel purified with a QIAquick PCR Purification Kit (QIAGEN), restrictions enzymes were supplied by NEB, PCR was performed using Phusion High-Fidelity DNA Polymerase (Thermo Fisher Scientific) and plasmids isolated with the PureYield Plasmid Midiprep System (Promega). Plasmids inserts were verified by sequencing (Microsynth AG). Plasmid maps are provided in Additional file 3: Figure S2.

### Transformation of *T. reesei* and genotyping

Transformation was performed by electroporation [66]. Transformants were purified via conidiospores on selective plates containing 0.1% (w/v) Triton X-100 in two rounds before genetic analysis. For selection 100 µg/mL hygromycin B (Sigma) or 50 µg/mL nourseothricin (Jena Bioscience GmbH) were added to PDA plates. The *T. reesei* QM9414 Δ*tku70* [64] was transformed with pK1. Expression of CEL12A from the P*cDNA1*-*cel12a* expression cassette in the transformants was verified by a carboxymethyl cellulose plate assay, compared to a *T. reesei* QM9414 *cel12a*^+^ reference strain [43] and strain K1 selected for further experiments.

*Rac1* was deleted in *T. reesei* K1. pDELrac1 was linearized with *Ssp*I (Thermo Fisher Scientific) and 10–15 µg DNA were transformed by electroporation. The homologous integration of the *rac1* deletion cassette was tested by PCR from genomic DNA, using the oligonucleotides Gen_DEL_rac1_fw and _rv. PCR of deletion strains resulted in a 3.45 kb band, whereas the parental strain showed a 2.99 kb band (data not shown). All primers for genotyping are listed in Additional file 2: Table S2.

### DNA and RNA extraction, reverse transcription and qPCR

For isolating DNA, mycelium was scratched off a PDA plate with a spatula and DNA was extracted according to a quick extraction protocol [67]. Biomass samples for RNA extraction from liquid cultures were filtered with a Miracloth filter, shock frozen with liquid nitrogen and stored at − 80 °C. For the RNA isolation the peqGOLD TriFast mix (PeqLab) was used according to the protocol. The RevertAid H Minus First Strand cDNA Synthesis Kit (Fermentas) was used for cDNA synthesis. All qPCRs were performed with the Luna Universal qPCR Master Mix (NEB). Results were evaluated with REST 2007 [68] freeware by QIAGEN. All primers for qPCR are listed in Additional file 2: Table S3.

### Determination of biomass, extracellular protein and enzyme activities

The biomass in the d-glucose and lactose liquid media was determined by filtering onto Glass Microfiber Filter GF/C, Diameter 47 mm (Whatman). After filtration, the filter was dried on 80 °C. In cellulose cultures the biomass was measured indirectly by the amount of internal protein. 1 mL culture broth was centrifuged for 30 min. The supernatant was discarded and the pellet was washed with distilled water. The pellet was resuspended in 1 mL 1 M NaOH and incubated for 2 h and 1000 rpm at room temperature. The suspension was centrifuged for 10 min and the protein content of the supernatant was determined by the Biorad protein assay reagent (BioRad).

Supernatants of liquid culture were filtered through a Miracloth filter and stored at − 20 °C. The protein of the lactose cultures supernatants was measured with the Pierce™ BCA Protein Assay Kit using the microtiter plate protocol (Thermo Fisher). The *endo*-1,4-β-d-glucanase activity (*endo*-cellulase) of the filtered supernatants from all carbon sources was determined by the Azo-CMC-Assay (Megazymes). The reaction was down-scaled to 200 µL aliquots of the supernatant. All reactions were performed in duplicates from biological triplicates in case of the reference K1 and quintuplicates in case of the deletion strains.

For a carboxymethyl cellulose activity assay on agar plates, transformants were grown on a defined medium agar plates (6 g/L (NH_4_)_2_SO_4_, 1 g/L MgSO_4_, 10 g/L sodium citrate, 20 mL/L trace element solution (see Mandels Andreotti medium), 10 g/L d-glucose, 15 g/L agar noble, pH 5.5) supplemented with 0.5% carboxymethyl cellulose and incubated for 8 h at 28 °C. Afterwards the agar plates were stained with a 0.2% Congo Red solution for 15 min, washed with 1 M NaCl and the clearing zone of each transformant resulting from the cellulase activity determined.

### Microscopy

Samples of three biological replicates of Δ*rac1* and three technical replicates of the K1 strain were each drawn from shake flask cultivations on MA medium with the respective carbon sources d-glucose, lactose or cellulose. For cellulose samples a cotton blue staining was applied: 1 µL cotton blue dye (Sigma-Aldrich) was added to 10 µL liquid culture, and incubated at room temperature for 5 min. The samples were examined with a Leica Microscope DMi8.

For hyphae characterisation, three replicates of K1 and Δ*rac1* were grown on MA + lactose for 27 h at 28 °C and 250 rpm. Mycelium was spread on a microscope slide and was examined with a Leica Microscope DMi8 with a 63× objective. The pictures were transferred to Image J, where length, diameter and branching frequencies were determined.

### Fed batch cultivation

A 100 mL preculture was grown on batch medium in a shake flask (10 g/L (NH_4_)_2_SO_4_, 4 g/L KH_2_PO_4_, 0.5 g/L MgSO_4_*7 H_2_O, 0.4 g/L CaCl_2_, 0.5 g/L lactose, 20 g/L d-glucose, aqueous extract from 40 g wheat bran, 7 mg/L FeSO_4_*7 H_2_O, 2 mg/L MnSO_4_*H_2_O, 7 mg/L ZnSO_4_*H_2_O, pH 4.0). The cultivation was started in 1 L batch culture with d-glucose as main carbon source (2%) and its depletion was monitored by CO_2_-generation. After the d-glucose levels decreased to 0.2 g/L, the batch was starved for another hour before the feeding with lactose began at a rate of 0.25 g/L*h from a 10% lactose solution. After 100 h of feeding, samples were drawn for determination of biomass formation and viscosity of the cultivation broth. The pH of 4 was regulated by addition of 12% w/v NH_4_OH, temperature was 28 °C, aeration rate was 0.5 vvm and the impeller speed was 1000 rpm. A silicon-based antifoam was used during feeding. For determination of the dry weight 1.8 mL of batch culture was drawn with a syringe and centrifuged for 10 min at 10,000 rpm in an Eppendorf tube. The supernatant was removed, the pellet was washed two times with 0.9% NaCl and dried at 100 °C for 24 h. QM9414 was measured in technical triplicates, ∆*rac1* was measured in biological triplicates and each biological in technical triplicates (Additional file 1: Figure S1).

### Viscosity measurements

Viscosity was measured with a Malvern Kinexus Lab + KNX2110 viscosimeter. The angular velocities were 0.9503, 1.196, 1.506, 1.896, 2.387, 3.005, 3.783, 4.763, 5.996, 7.549 and 9.503 rad/s. The angular velocities were integrated and normed to *T. reesei* QM9414.

## Supplementary information

**Additional file 1: Figure S1.** Bioreactor process data of fed batch cultivations.

**Additional file 2.** Primers used in this study. **Table S1.** Primers for vector cloning. **Table S2.** Primers for *T. reesei* genotyping. **Table S3.** Primers for qPCR with primer efficiency.

**Additional file 3: Figure S2.** Plasmid maps of the vectors.

## Data Availability

All data generated or analysed during this study are included in this published article and its Additional files.
