# Supplementary material for: Deletion of the small GTPase rac1 in Trichoderma reesei provokes hyperbranching and impacts growth and cellulase production
Source: Fungal Biol Biotechnol. 2019 Oct 18;6:16. doi: 10.1186/s40694-019-0078-5 (PMC6798449; doi:10.1186/s40694-019-0078-5)
Supplement: Supplementary file 1 — Additional file 1: Figure S1. Bioreactor process data of fed batch cultivations. [file 40694_2019_78_MOESM1_ESM.docx]

**Additional file 1**


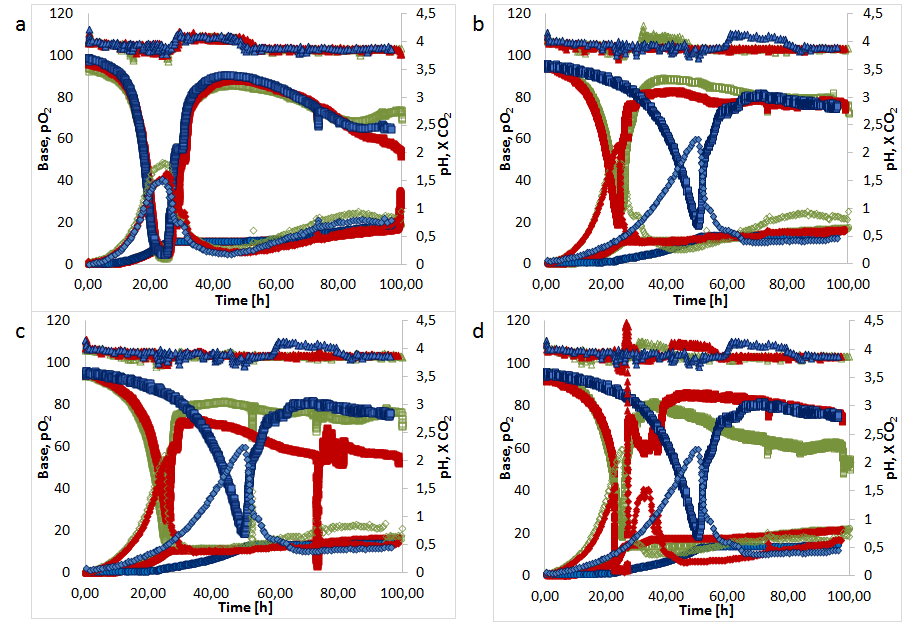


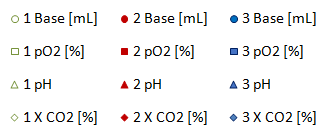


**Figure S1 Bioreactor process data of fed batch cultivations.** Recording of the process data: used base (12 % w/v NH_4_OH) in mL, pH, partial pressure of the dissolved oxygen pO_2_ in % and molar fraction of carbon dioxide in the offgas X CO_2_ in % over cultivation time in h of a) 9414 in technical triplicates b) rac1 transformant 1 in technical triplicates c) rac1 transformant 2 in technical triplicates d) rac1 transformant 3 in technical triplicates.
