# Supplementary material for: Deletion of the small GTPase rac1 in Trichoderma reesei provokes hyperbranching and impacts growth and cellulase production
Source: Fungal Biol Biotechnol. 2019 Oct 18;6:16. doi: 10.1186/s40694-019-0078-5 (PMC6798449; doi:10.1186/s40694-019-0078-5)
Supplement: Supplementary file 2 — Additional file 2. Primers used in this study. Table S1. Primers for vector cloning. Table S2. Primers for T. reesei genotyping. Table S3. Primers for qPCR with primer efficiency. [file 40694_2019_78_MOESM2_ESM.docx]

Table S1 Primers for cloning of vectors

| M13_fw | gtaaaacgacggccagt |
| --- | --- |
| M13_rv | ACAGGAAACAGCTATGACC |
| nat1_BamHI_fw4 | CTCCTACTTCAACCACAACCGGATCCATGACCACCCTCGACGACACGGC |
| nat1_EcoRI_rv4 | CTGCTGGCGCATATAGGACACGAATTCAGGGGCAGGGCATGCTCATG |
| Ppgi_XbaI_fw4 | GCGCTCTAGACTTTCCCGGCACTCGTCGT |
| Ppgi_BamHI_nat1_rv | GCCGTGTCGTCGAGGGTGGTCATGGATCCGGTTGTGGTTGAAGTAGGAG |
| Pcdna1_fw | ctcgagcagacaatgatggtagc |
| Pcdna1_rv | atcgatgagagaagttgttggattgatc |
| cel12a_fw | TAGCGTCGCAATGAAGTTC |
| cel12a_rv | atcgatTGTCTAATCGACGTGCA |
| Inf_pK1_NtR_fw | AGTGAGTCGTATTACAATTCataaccatcgcatgcaaggtc |
| Inf_pK1_NtR_rv | AATTTCGAGCTTGGCGTAATGGTACCGAGCTCGAATT |
| Inf_NtR_pK1_fw | atactcgccaccccagccggATTACGCCAAGCTCGAAATTAA |
| Inf_NtR_pK1_rv | ACCTTGCATGCGATGGTTATGAATTGTAATACGACTCACTATAGG |
| Inf_pUC19_rac1_fw | attcgagctcggtacccgggGGAGCGTTTCAAAGCAGC |
| Inf_pUC19_rac1_rv | cctgcaggtcgactctagagCAACGGAAAGTCGGAGGAC |
| Inf_DEL_rac1_fw | gacaacacatgggaaacaagGATAGGGATGCAGCAATCC |
| Inf_DEL_rac1_rv | acctagtacacacaatcgccGGAGGAGAGGGTTTCAAG |
| HmR_fw | ggcgattgtgtgtactaggt |
| HmR_rv | cttgtttcccatgtgttgtctga |

Table S2 Primers for *T. reesei* QM9414 genotyping

| Gen_DEL_rac1_fw | CTCCATTGCCCTGTTCCCAT |
| --- | --- |
| Gen_DEL_rac1_rv | GAGCCGGACACATAGACGAG |

Table S3 Primers for qPCR with primer efficiency.

| qPCR_tef1_fw | CCACATTGCCTCCAAGTTCGC | 87%, R2 = 0,994 |
| --- | --- | --- |
| qPCR_tef1_rv | GTCGGTGAAAGCCTCAACGCAC |  |
| qPCR_cel7a_fw | CCGAGCTTGGTAGTTACTCTG | 94%, R2 = 0,997 |
| qPCR_ cel7a _rv | GGTAGCCTTCTTGAACTGAGT |  |
| qPCR_cel12A_fw  qPCR_cel12A_rv | CAGCCTCACCATGAACCAGT  CGTTGAGCTTCAGCATCACG | 97%, R^2^ = 0,988 |
| qPCR_sar1_fw | TGGATCGTCAACTGGTTCTACGA | 90%, R2 = 0,950 |
| qPCR_sar1_rv | GCATGTGTAGCAACGTGGTCTTT |  |
| qPCR_actin_fw | TGAGAGCGGTGGTATCCACG | 101 %, R2 = 0,995 |
| qPCR_actin_rv | GGTACCACCAGACATGACAATGTTG |  |
| qPCR_rac1_fw | TCTGTTTCTCCGTCGTGAGC | 90%, R2 = 0,991 |
| qPCR_rac1_rv | GAGTTCCAGACGAGTGGTGG |  |
| qPCR_cdc42_fw | AGCGCGTTGACTCAGTACAA | 82%, R2 = 0,952 |
| qPCR_cdc42_rv | TGGGATTTCTTCTTGGGGGC |  |
