# Supplementary material for: Deletion of the small GTPase rac1 in Trichoderma reesei provokes hyperbranching and impacts growth and cellulase production
Source: Fungal Biol Biotechnol. 2019 Oct 18;6:16. doi: 10.1186/s40694-019-0078-5 (PMC6798449; doi:10.1186/s40694-019-0078-5)
Supplement: Supplementary file 3 — Additional file 3: Figure S2. Plasmid maps of the vectors. [file 40694_2019_78_MOESM3_ESM.docx]

**Additional file 3**


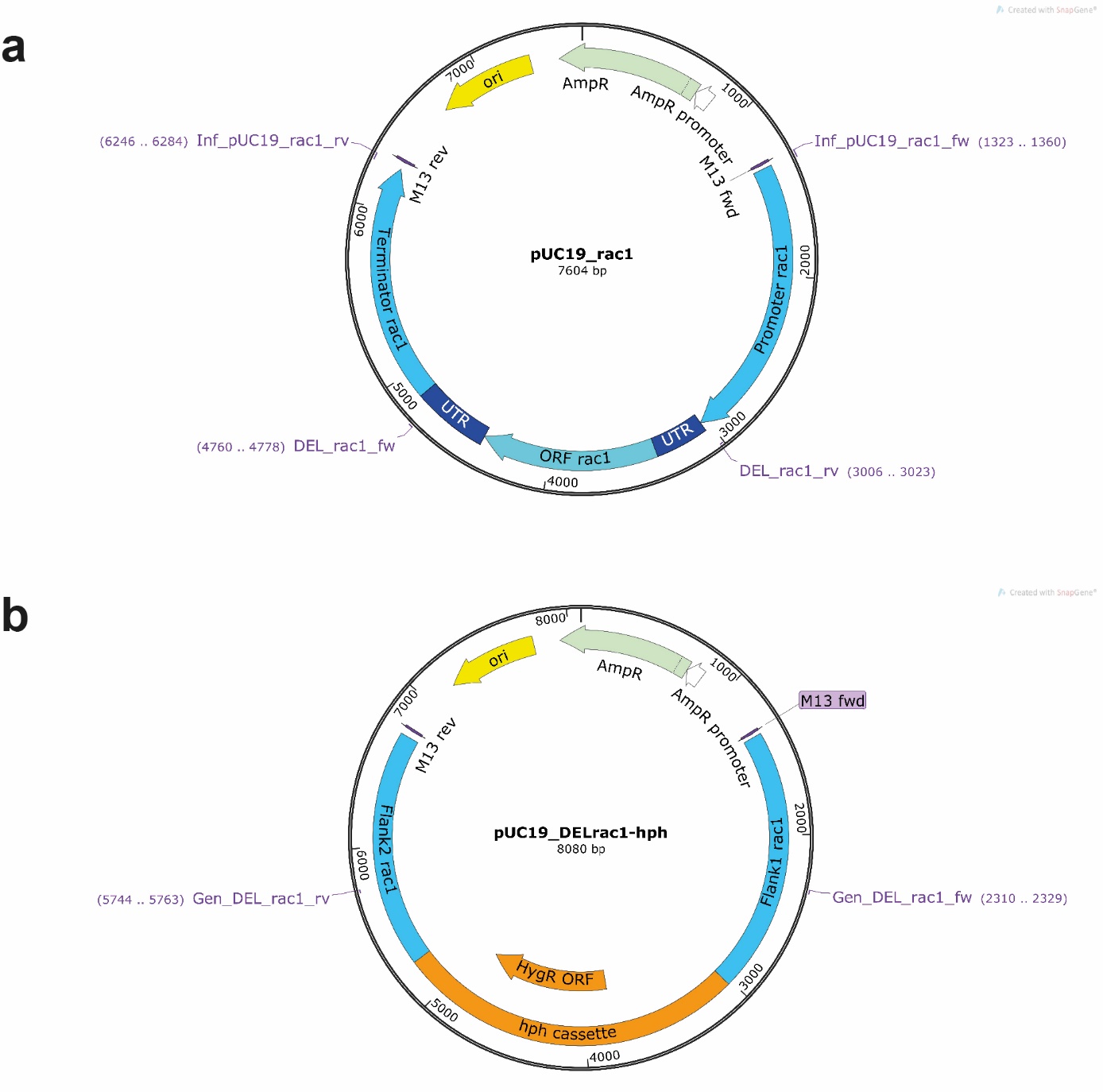


Figure S2 Plasmid maps of the vectors. a) *rac1* from *T. reesei* QM9414 was amplified from the genome with Inf_pUC19_rac primers and inserted into the BamHI site of pUC19, including promoter and terminator. b) The *rac1* ORF was deleted by PCR of the backbone with DEL_rac1 primers and substituted by a *hph* cassette, coding for a hygromycin resistance. The marked Gen_DEL_rac primers were used for insertion control.
